# Real-Time Analysis of Temperature Changes in Composite Increments and Pulp Chamber during Photopolymerization

**DOI:** 10.1155/2015/923808

**Published:** 2015-10-18

**Authors:** Ryan Jin-Young Kim, In-Bog Lee, Jin-Young Yoo, Su-Jung Park, Sin-Young Kim, Young-Ah Yi, Ji-Yun Hwang, Deog-Gyu Seo

**Affiliations:** ^1^Department of Conservative Dentistry and Dental Research Institute, School of Dentistry, Seoul National University Seoul 110-749, Republic of Korea; ^2^Department of Dental Hygiene, Wonju College of Medicine, Yonsei University, Wonju 220-701, Republic of Korea; ^3^Department of Conservative Dentistry, Seoul St. Mary's Dental Hospital, Catholic University of Korea, Seoul 137-701, Republic of Korea; ^4^Department of Dentistry, Inje University Seoul Paik Hospital, Seoul 411-706, Republic of Korea; ^5^Nutrition Education Major, Graduate School of Education, Sangmyung University, Seoul 110-743, Republic of Korea

## Abstract

*Objective*. The aim of this study was to evaluate the temperature change at various sites within the composite and on the pulpal side of dentin during polymerization of two composite increments. *Materials and Methods*. Class I cavities prepared in third molars were restored in two composite increments (*n* = 5). Temperatures were measured for 110 s using eight thermocouples: bottom center of cavity (BC), top center of 1st increment (MC), top center of 2nd increment (TC), bottom corner of cavity (BE), top corner of 1st increment (ME), top corner of 2nd increment (TE), pulpal side of dentin (PD), and center of curing light guide tip (CL). *Results*. Maximum temperature values (°C) measured during polymerization of 1st increment were MC (59.8); BC (52.8); ME (51.3); CL (50.7); BE (48.4); and PD (39.8). Maximum temperature values during polymerization of 2nd increment were TC 58.5; TE (52.6); MC (51.7); CL (50.0); ME (48.0); BC (46.7); BE (44.5); and PD (38.8). *Conclusion*. Temperature at the floor of the cavity was significantly higher during polymerization of 1st increment compared to 2nd increment. Temperature rise was higher at the center than at the corner and at the top surface than at the bottom surface of each increment.

## 1. Introduction

Composite resins have become the most widely used direct restorative materials in meeting the patient demand for esthetic dentistry. Despite the many advantages of composites, heat is inevitably generated during photopolymerization by the exothermic reaction of composite resin and by the light-curing unit* per se* [1]. It is well known that excessive heat is detrimental to living tissues such as the dentin-pulp complex and periodontal tissue. Possible outcomes of thermal injuries during dental procedures include transient pulpal inflammation, irreversible pulpal inflammation or necrosis [2, 3], bone resorption, and tooth ankylosis [4].

Numerous studies have demonstrated a positive correlation between the light-curing unit intensity and temperature rise [5–11]. The degree of dentin mineralization [12] and the remaining dentin thickness [12–14] showed a negative correlation with a temperature rise during polymerization of light-cured composites. Previous studies have evaluated the temperature rise during polymerization of composite resin by using a thermistor [5, 9], thermocouples [6–8, 10, 12, 15], differential scanning calorimetry [16], differential thermal analysis [17, 18], and infrared thermography [11, 14].

However, these studies measured the temperature change only at the bottom surface of composites [5, 6, 9], at the pulpal surface of dentin [7, 10, 12], or at the center of composites [8, 15]. Recently, Chang et al. [19] measured the polymerization temperature at multiple spots along the external surface of a Class II cavity according to the curing depth and approximation to the cavity wall using infrared thermography. Nevertheless, to date there is no published study that has performed the simultaneous measurements of the temperature changes within the composite resin and at the pulpal side of the dentin using human teeth under simulated* in vivo* conditions. Moreover, no studies exist evaluating the effect of incremental curing of composites on the temperature changes.

The aim of this paper was to compare the temperatures measured at various sites along the bottom and top surfaces of composite increments and the pulpal side of dentin during photopolymerization of two increments of composites in real-time using multiple thermocouples.

## 2. Materials and Methods

### 2.1. Specimen Preparation

**Figure 1:**
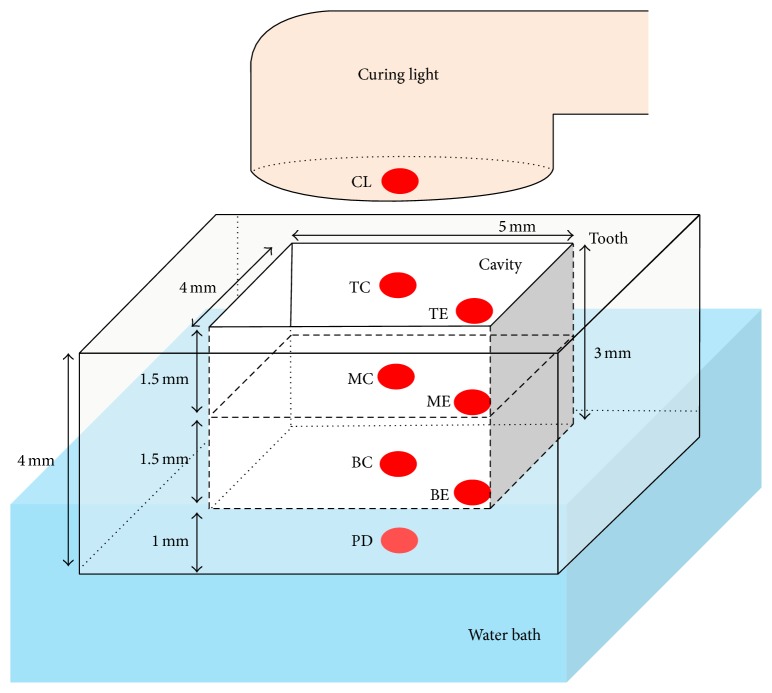
Schematic diagram of specimen setup showing the position of thermocouples and dimension of tooth cavity (BC: bottom center of the cavity; MC: top center of 1st increment; TC: top center of 2nd increment; BE: bottom corner of 1st increment; ME: top corner of 1st increment; TE: top corner of 2nd increment; PD: bottom center on pulpal aspect of dentin; and CL: center of curing light guide tip).

The experimental setup is shown in Figure 1. Five freshly extracted, intact caries-free third molars stored in 0.5% chloramine-T solution were used for the study. The occlusal surface of each tooth was ground to a flat surface and prepared with Class I cavities (mesiodistal length, 5 mm; buccolingual width, 4 mm; and depth, 3 mm) using a flat end cylindrical diamond bur. The lower portion of the crown was horizontally sectioned at 4 mm below the occlusal surface of the cavity, leaving a 1 mm thick dentin remaining between the internal bottom surface of the cavity and the external horizontally sectioned surface.

The temperature measurement sites are as follows, assigned according to the position of the thermocouples:

 BC: bottom center of the cavity,
 MC: top center of the 1st increment composite resin,
 TC: top center of the 2nd increment composite resin,
 BE: bottom corner of the 1st increment composite resin,
 ME: top corner of the 1st increment composite resin,
 TE: top corner of the 2nd increment composite resin,
 PD: bottom center on the pulpal aspect of dentin,
 CL: center of the curing light guide tip.

Two vertical grooves (one for the center and another for the corner positions) extending from the occlusal surface to the bottom of the cavity were made for each tooth by cutting the corners of the teeth using a diamond bur to accommodate K-type thermocouples with a diameter of 0.5 mm (TT-K-36, Omega, Stamford, CT, USA) within the cavity. A flowable composite resin (Charisma, Shade A2, Lot 10128, Heraeus-Kulzer, Hanau, Germany) was applied around the thermocouple wires within the grooves and light-cured for 20 s to secure the thermocouples, which were connected to a thermocouple conditioner and setpoint controller (AD597, Analog Devices, Norwood, MA, USA). The thermocouple signals were digitized in real-time with a data acquisition board (cDAQ-9174, National Instruments, Austin, TX, USA) equipped with an analog input module (NI 9205, National Instruments) using a data acquisition software (LabVIEW, National Instruments).

### 2.2. Temperature Measurement

Temperature changes were recorded during polymerization of two increments of composite resin (Filtek Z250, Shade A2, Lot N506344, 3M ESPE, St. Paul, MN, USA). For temperature measurement, thermocouples were position at BC, BE, PD, and CL, and then after the cavity was filled with the 1st increment (1.5 mm thick, 30 mm^3^), additional thermocouples were placed at MC and ME. A modified periodontal probe with a marking at 1.5 mm from the tip was positioned along the axis of the tooth cavity in order to control the thickness of the composite increment. A baseline was obtained for 10 s without light exposure, after which the curing light was turned on for 20 s at 750 mW/cm^2^ (Elipar S10 LED curing light, 3M ESPE). The tip of the curing light was positioned 2 mm away from the specimen. After recording the temperatures once every second for 110 s, a subsequent 2nd increment of composite (1.5 mm thick, 30 mm^3^) was placed over the light-cured 1st increment up to the top of the cavity, and additional thermocouples were placed at TC and TE. As in the 1st increment, the temperatures were recorded for 110 s, including a 10 s baseline and a 20 s light-curing. Temperatures were measured at six sites except TC and TE for the 1st increment and at all eight sites for the 2nd increment. During temperature measurements, each specimen was secured in a water bath maintained at 36.5°C, whereby the 1 mm thick remaining dentin was immersed in water with the rest of the tooth above the water level. Each experimental condition was replicated five times.

### 2.3. Statistical Analysis

The mean maximum temperatures of the assigned measurement sites were analyzed using one-way ANOVA followed by Tukey's HSD post hoc comparison analysis. The analyses were conducted with SPSS software version 21 (IBM, New York City, NY, USA) at a significance level of 0.05.

## 3. Results

**Figure 2:**
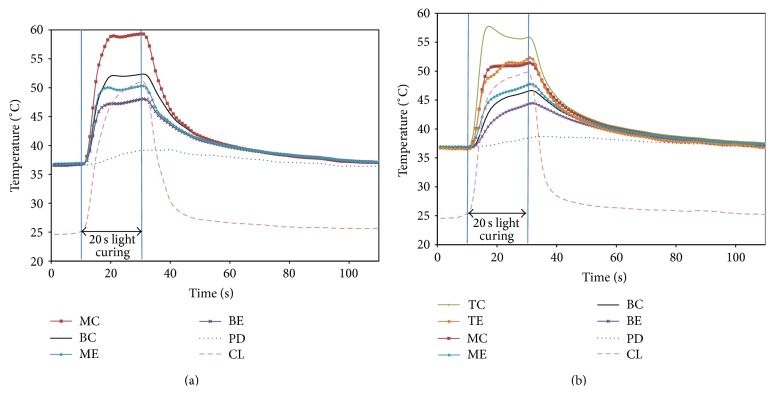
Mean temperatures measured during polymerization of (a) 1st composite increment and (b) 2nd composite increment (BC: bottom center of the cavity; MC: top center of 1st increment; TC: top center of 2nd increment; BE: bottom corner of 1st increment; ME: top corner of 1st increment; TE: top corner of 2nd increment; PD: bottom center on pulpal aspect of dentin; and CL: center of curing light guide tip).

Mean temperatures as a function of time during polymerization of the 1st and 2nd increments are shown in Figure 2. A rapid increase and decrease in temperature corresponded to the curing light being turned on and off, except at PD.

**Figure 3:**
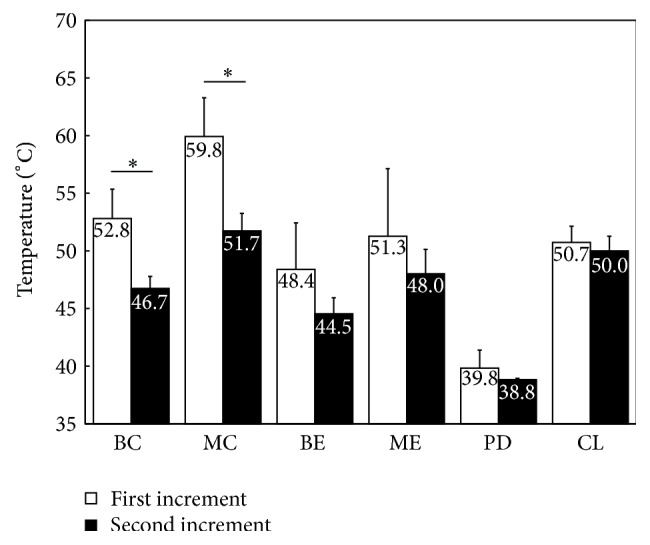
Means and standard deviations of maximum temperature during polymerization of 1st and 2nd composite increments. Asterisk indicates statistical difference between the 1st and 2nd composite increments (BC: bottom center of the cavity; MC: top center of 1st increment; TC: top center of 2nd increment; BE: bottom corner of 1st increment; ME: top corner of 1st increment; TE: top corner of 2nd increment; PD: bottom center on pulpal aspect of dentin; and CL: center of curing light guide tip).

The mean maximum temperatures during polymerization of the 1st and 2nd composite increments are summarized in Table 1. The maximum temperatures were significantly increased with a decrease in distance between the curing light and the measurement site. Also, the maximum temperatures were significantly higher at the center when compared to the corner of the composite within the same depth. During polymerization of the 2nd composite increment, the maximum temperatures measured at BC and MC were significantly lower than those obtained during the initial curing of the 1st increment (*P* < 0.05) (Figure 3). The pulpal aspect of the dentin at PD exhibited the lowest mean maximum temperatures of 39.8 ± 1.5°C during polymerization of the 1st increment and 38.8 ± 0.2°C during polymerization of the 2nd increment. The maximum temperatures recorded at MC (59.8 ± 3.2°C) and TC (58.5 ± 2.1°C) were highest of all the measurement sites during polymerization of the 1st increment and 2nd increment, respectively (*P* < 0.05).

## 4. Discussion

The temperature rise of light-cured composites is typically caused by both an exothermic reaction process and the transmission of heat from the curing light itself [1]. An exothermic reaction takes place as carbon-to-carbon double bonds in the monomer change to a single bond during polymerization [11, 14]. Previous studies have reported that the temperature rise is greater with increasing power density [5–8, 10] and irradiation time [9, 10] of the curing light and with decreasing the distance from the curing light tip [9].

In the present study, multiple thermocouples were used to provide a comprehensive understanding of temperature changes at various sites within the cavity and at the pulpal aspect of dentin during polymerization of two increments of composites. Within the cavity, temperatures were measured at the center as well as the corner of each increment since the temperature change in the corner is also of value to note considering the tooth is a living tissue; the corner adjacent to the cavity wall is in communication with the dentin-pulp complex via odontoblasts and their processes through the interconnecting dentinal tubules.

At the same depth within the composite increments, the temperature rise at the center was higher than at the corner adjacent to the cavity walls. It could be explained by the difference in the amount of reacted composites. Studies have shown that there is a proportional relationship between the temperature rise from the exothermic reaction and the amount of composite resin [11, 14]. In this study, the thermocouples placed at the center were surrounded by a much greater amount of composites to be polymerized, which in turn resulted in a higher temperature rise due to greater heat generation via the exothermic reaction, than those placed in the peripheral composites. Considering the greater heat generation with increasing amount of composite resin to be polymerized, clinicians should consider placing a smaller amount of composite resin for the initial first layer in order to minimize potential thermal irritation to the pulp.

An inversely proportional relationship was found between the temperature rise and distance from the curing light to the measurement sites in each incremental layer of composite resin. The top surface received the maximum energy from the curing light as it was closer to the curing light. Al-Qudah et al. [11] have reported that the temperature rise on the lower surface of the composite was lower with an increase in the thickness of the composite specimens. This observation can be explained by the exponential reduction in the light intensity, due to light scattering, with increasing composite thickness [20]. Thus, as the light passes through the thicker composite, the effectiveness of the light cure at the bottom surface is impaired, as evidenced by the reduction in the degree of conversion [17, 21]. Such a pattern of temperature rise emphasizes the important role the distance from the light source and thickness of composite material have in the temperature rise. Knežević et al. [22] also showed a higher degree of conversion and temperature rise on the surface than at a depth of 1 mm, supporting the current findings of a higher temperature on the top surface of each increment. A light-emitting diode (LED) curing light was employed in the present study to help clinicians understand how much the temperature may rise during a composite restoration in the clinical setting using the currently most commonly used curing light type for photopolymerization of the composite. With regard to the type of light-curing units, LED curing units are reported to generate less heat as a result of having narrower emission spectra, compared to halogen curing units, which have broad emission spectra far into the ultraviolet A range [23].

As the curing light was turned on and off, a corresponding rapid increase and decrease in temperature was observed at all measurement sites except the pulpal side of the dentin. The lowest temperature rise was observed after a latent phase of approximately 10 s at the pulpal side of dentin during polymerization of both increments (Figure 2). The surface temperature on the pulpal aspect of the dentin was further reduced from 39.8 ± 1.5°C (3.3°C rise) in the 1st increment to 38.8 ± 0.2°C (2.3°C rise) in the 2nd increment. Zach and Cohen [2] evaluated the histological responses of dental pulp to thermal stress in Macaca rhesus monkeys. They found 15% of the healthy teeth failed to recover when the pulp temperature was increased by 5.6°C, 60% after an increase of 11.1°C, and almost 100% when the temperature was raised above 11.1°C. Thus, an increase of intrachamber temperature by 5.6°C has been considered a critical threshold for irreversible pulpal damage [2]. As per the current results, the temperature rise in the pulpal aspect of dentin, underneath the 1 mm thick remaining dentin, during polymerization of both increments was below the critical value. Since the dentin is known to be an excellent thermal insulator [14], having a 2.5-fold lower thermal diffusivity than enamel [24], the temperature rise was significantly reduced and slowed down on the pulpal side of the dentin. The remaining dentin thickness is therefore an important protective factor against thermal injury to the pulp [12–14]. In addition to the insulating effect of dentin, the cooling effect of water within the pulpal chamber reduced the temperature rise on the pulpal side of the dentin. Temperature rise would be even greater, increasing the potential for jeopardizing pulpal health during composite restoration, in the following situations where the remaining dentin thickness is reduced: (1) when more remaining mineralized dentin [12] associated with caries or previous treatment history exists due to the presence of greater amount of reparative dentin that has a higher thermal diffusivity; (2) when local anesthesia including vasoconstrictor is administered, reducing the cooling effect of pulpal blood due to the reduction of pulpal blood flow [25, 26]; and (3) when the volume of the pulp chamber is reduced, especially in aged people, thereby relatively reducing the cooling potential.

The present study demonstrated that the values and patterns of temperature changes were different among various sites within the composite during photopolymerization. Further studies are required to evaluate the effect of light-curing intensity and mode, remaining dentin thickness, and thickness of each incremental composite resin layer on temperature rise, thereby providing clinical recommendation in the context of minimizing temperature rise.

## 5. Conclusions

Temperature measured at the floor of the cavity was higher during polymerization of the 1st increment. The temperature rise was higher at the center than the corner and at the top surface than at the bottom surface of each increment. Given the higher temperature rise with the increasing amount of composites, it is suggested to avoid placing a large amount of the composite as a first layer to minimize potential thermal damage to the pulp, especially in the case where the remaining dentin is thin. Within the limitations of this experiment, the temperature rise on the pulpal aspect of dentin underneath the 1 mm thick remaining dentin does not seem to cause detrimental effect on the pulpal health.

## Figures and Tables

**Table 1:** Mean maximum temperatures (°C) at assigned measurement sites during polymerization of each increment.

	Measurement sites							
	BC	MC	TC	BE	ME	TE	PD	CL
1st increment	52.8 (2.5)^b^	59.8 (3.8)^a^	N/A	48.4 (4.0)^b^	51.3 (5.9)^b^	N/A	39.8 (1.6)^c^	50.7 (1.4)^b^
2nd increment	46.7 (1.1)^df^	51.7 (1.5)^bc^	58.5 (2.1)^a^	44.5 (1.4)^ef^	48.0 (2.1)^cde^	52.6 (4.0)^b^	38.8 (0.1)^g^	50.0 (1.3)^bd^

Standard deviations are in parentheses.

Values with identical superscript letters are similar within the same increment (Tukey's HSD, *P* > 0.05).

N/A: Not applicable.

BC: bottom center of the cavity; MC: top center of 1st increment; TC: top center of 2nd increment; BE: bottom corner of 1st increment; ME: top corner of 1st increment; TE: top corner of 2nd increment; PD: bottom center on pulpal aspect of dentin; and CL: center of curing light guide tip.
